# Dynamics of a model for the degradation mechanism of aggregated α-synuclein in Parkinson's disease

**DOI:** 10.3389/fncom.2023.1068150

**Published:** 2023-04-13

**Authors:** Bojie Yang, Zhuoqin Yang, Lijie Hao

**Affiliations:** ^1^School of Mathematical Sciences and LMIB, Beihang University, Beijing, China; ^2^School of Mathematics Science, Tianjin Normal University, Tianjin, China

**Keywords:** Parkinson's disease, α-synuclein, autophagy lysosome pathway, mTOR, tri-stability

## Abstract

Accumulation of the misfolded synaptic protein α-synuclein (αSyn^*^) is a hallmark of neurodegenerative disease in Parkinson's disease (PD). Recent studies suggest that the autophagy lysosome pathway (ALP) including both the Beclin1-associated and mTOR-signaling pathways is involved in the αSyn^*^ clearance mechanism. In this study, a mathematical model is proposed for the degradation of αSyn^*^ by ALP with the crosstalk element of mTOR. Using codimension-1 bifurcation analysis, the tri-stability of αSyn^*^ is surveyed under three different stress signals and, in addition, consideration is given to the regulatory mechanisms for the Beclin1- and mTOR-dependent rates on αSyn^*^ degradation using the codimension-1 and−2 bifurcation diagrams. It was found that, especially under internal and external oxidative stresses (*S*_1_), the bistable switch of the aggregation of αSyn^*^ can be transformed from an irreversible to a reversible condition through the ALP degradation pathways. Furthermore, the robustness of the tri-stable state for the stress *S*_1_ to the parameters related to mTOR-mediated ALP was probed. It was confirmed that mTOR-mediated ALP is important for maintaining the essential dynamic features of the tri-stable state. This study may provide a promising avenue for conducting further experiments and simulations of the degradation mechanism of dynamic modeling in PD.

## Introduction

Parkinson's disease (PD) is the second most frequent neurodegenerative disease, after Alzheimer's disease, with an incidence rate among humans as high as 2% after 65 years of age (Goedert et al., 2013; Bridi and Hirth, 2018). From a neuropathological point of view, the hallmark of PD is the loss of dopaminergic neurons in the substantia nigra pars compacta (SNc). It is most likely caused by the presence of neuronal cytoplasmic inclusions called Lewy bodies (LBs) due to abnormal accumulation of α-synuclein (αSyn) (Maries et al., 2003; Ruiperez et al., 2010; Gallegos et al., 2015; Choi et al., 2020).

Increased levels of αSyn have been demonstrated to cause the loss of mitochondria electron transport chain complex I activity resulting in increased reactive oxygen species (ROS) (Cali et al., 2011; Schapira and Jenner, 2011; Blesa et al., 2015; Scialo et al., 2017). Subsequently, excessive levels of ROS result in the upregulation of misfolded αSyn (αSyn^*^) and damage to dopaminergic neurons in PD (Kolodkin et al., 2020; Thorne and Tumbarello, 2022). Cloutier and Wellstead (2012) have proposed a double positive feedback loop for the mutual promotion between ROS and αSyn^*^ within a PD model. The model contains multiple complex interactions involving molecular pathways and cellular processes, and hence the model has been further simplified to highlight the central feedback motif of αSyn^*^ and ROS with stress signals (Cloutier et al., 2012).

Autophagy is a “self-eating” process arising through the digestion of cellular components (Komatsu et al., 2007; Harris and Rubinsztein, 2011; Zhao et al., 2015; Fussi et al., 2018; Sotthibundhu et al., 2018). The upregulation of the autophagy lysosome pathway (ALP) promoting the degradation of αSyn^*^ is a very important degradation mechanism for maintaining homeostasis in the pathology of PD (Spencer et al., 2009; Vilchez et al., 2014; Erustes et al., 2018; Malik et al., 2019; Bekker et al., 2021). However, the aforementioned models of the central feedback structure of ROS and αSyn^*^ do not allow for the vital clearance mechanisms of ALP to degrade αSyn^*^.

The mammalian target of rapamycin (mTOR) is a central regulator and modulates multiple aspects of ALP; hence, it is a novel therapeutic target for PD (Bove et al., 2011; Jiang et al., 2013; Xu et al., 2014; Zhu et al., 2019). The activation of mTOR is known to inactivate Beclin1-induced autophagy and promote Caspases-induced apoptosis under stress (Li et al., 2011; Siddiqui et al., 2015; Tavassoly et al., 2015; Cooper, 2018; Lu et al., 2020). Recent research suggests a complex network of mTOR signaling pathways implicated in the control of ALP which determines cell life and death (Sarkar et al., 2009; Jung et al., 2010; Kondratskyi et al., 2013; Shen et al., 2013). In consequence, the crosstalk element mTOR which regulates ALP to degrade the aggregation of αSyn^*^ may become one of the most crucial targets for the treatment of PD (Ebrahimi-Fakhari et al., 2014; Lan et al., 2017).

The aggregation of αSyn^*^ will trigger an appropriate endoplasmic reticulum (ER) stress response to decide cell survival or death (Kim et al., 2008; Cybulsky, 2017; Gomez-Suaga et al., 2018; Ren et al., 2021). Under tolerable stress conditions, autophagy plays a clearance role in removing aggregated αSyn^*^ for maintaining neuro homeostasis (Booth et al., 2014; Liu et al., 2018). However, under conditions of too long or too excessive stress levels, autophagy will switch to apoptosis as a “self-killing” pathway for better adaptation to the living environment (Heath-Engel et al., 2008; Djavaheri-Mergny et al., 2010; Wu et al., 2014; Chung et al., 2018). Kapuy et al. (2014) have presented a mathematical model that contains an interplay between major autophagy and apoptosis proteins mediated by mTOR. In that study, Beclin1, as the main protein regulator of autophagy, is activated by the endoplasmic reticulum stress sensor (ERS), and Caspases, as a primary inducer of apoptosis, is also activated by ERS.

In the present study, we seek to establish a mathematical model of mTOR-mediated ALP as the major protein clearance for the degradation of aggregated αSyn^*^. Two key points will be addressed: (1) the aggregation of αSyn^*^ promoted by ROS triggers the activation of ERS and then ALP and Caspases-dependent apoptosis and (2) mTOR crosstalk controls ALP with respect to the degradation of the aggregated αSyn^*^. Abundant non-linear dynamic analysis in the model expounds the tendency of protein concentrations to play an important role in clarifying whether the disease emerges or not.

**Figure 1:**
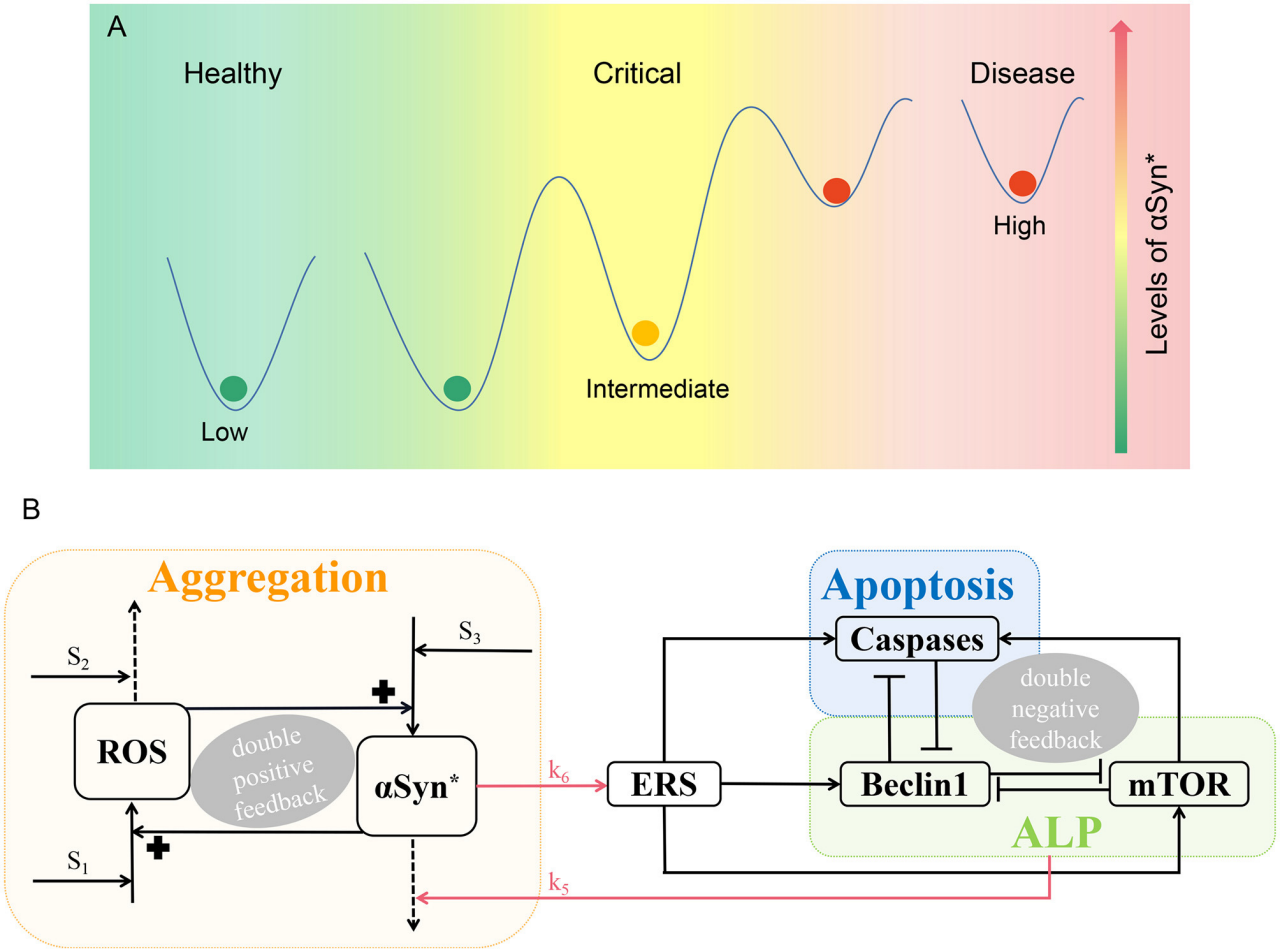
Schematic illustration of PD. **(A)** According to the levels of αSyn*, there are three different states, i.e., the low, intermediate, and high steady states parallel to the healthy, critical, and disease states, respectively. **(B)** A three-module model network characterizing aggregation, ALP, and apoptosis. The promotion, degradation, and inhibition are denoted by solid lines with arrowheads, dotted lines with arrowheads, and blocked end lines, respectively.

Furthermore, recent studies show that in neurodegenerative disease, it is quite possible that a critical state, as evidenced by the existence of a tipping point, exists between healthy and disease states (Liu R. et al., 2019; McClellan and King, 2021). In this study, we seek to clarify the existence of healthy, critical, and disease states in the pathology of PD, based on the presence of low, intermediate, and high steady-state levels of αSyn^*^, respectively, as depicted in Figure 1A. Therefore, in the context of the tri-stability of αSyn^*^, it is considered noteworthy to study critical states to gain a better understanding of the pathogenesis of PD.

To investigate the characteristic of αSyn^*^ with tri-stability, the focus is given to the key molecules Beclin1 and mTOR in ALP which degrade αSyn^*^ under three different stresses, i.e., internal and external oxidative stresses (*S*_1_), age-related anti-oxidative mechanisms (*S*_2_), and the influence of genetic damage (*S*_3_) through codimension-1 bifurcation diagrams. Moreover, bifurcation analysis is used to survey the tri-stability under the three stresses controlled by the ALP-dependent degradation rates. In addition, all possible steady-state regions are explored by codimension-2 bifurcation analyses as well as the effect of the steady-state levels of αSyn^*^ under different initial conditions. We find that ALP related to Beclin1 and mTOR correlates to the bistable state which switches from the irreversible to the reversible state for the stress signal *S*_1_, which may have significant implications for the pathogenesis of PD. Finally, we investigate the robustness of the tri-stable state with respect to the mTOR-associated regulatory mechanisms. Overall, our findings provide new insights into the complex tri-stability dynamics as being a key regulatory mechanism where ALP mediated by mTOR degrades αSyn^*^ in PD.

## Methods

### Model of ALP regulated by mTOR for degradation of αSyn^*^

The mathematical network model is divided into three functional modules with Aggregation (orange), ALP (green), and Apoptosis (blue) illustrated in Figure 1B.

Aggregation is represented by a positive feedback loop between αSyn^*^ and ROS under three stresses *S*_1_, *S*_2_, and *S*_3_. Internal or external oxidative stress (such as nutrient starvation, mitochondria dysfunction, and the loss of dopamine) *S*_1_ promotes ROS, while stress *S*_2_ with mechanisms of age-related anti-oxidative (such as reduced energy metabolism and anti-oxidative capability) degrade ROS. Stress *S*_3_ which is characterized by having damaged protein mechanisms for over-expression that promotes the formation of αSyn^*^ (Cloutier et al., 2012).

Endoplasmic reticulum stress activated by αSyn^*^ (upper red arrow) promotes the activation of Beclin1-dependent autophagy *via* ALP, Caspases-dependent apoptosis, and mTOR (Kapuy et al., 2014). αSyn^*^ is aggregated through ALP (lower red arrow) including both mTOR signaling and Beclin1-associated pathways with protein clearance mechanisms. Three double-negative feedback loops are formed by mutual inhibitions between Beclin1 and Caspases, between Beclin1 and mTOR, and among Beclin1, Caspases, and mTOR when the activated mTOR promotes Caspases.

### Dynamic equations

The model is formulated as a coupled system of non-linear ordinary differential equations (ODEs) (1)–(6), which includes six components: the concentrations of [ROS], [αSyn^*^], [ERS], [mTOR], [Beclin1], and [Caspases].

The double-positive feedback loops between [ROS] and [αSyn^*^] are described in Equations (1) and (2). The first term in Equation (1) represents [ROS] generated by the background synthesis, stimuli *S*_1_, and [αSyn^*^]. The second term represents [ROS] degraded by stimuli *S*_2_. The dynamics of aggregated αSyn^*^ in Equation (2) includes [ROS] which promotes the generation of αSyn^*^ within the first term, and the basal and Beclin-1-dependent removal within the second term. There are two terms in Equation (3), the first term corresponds to [ROS]-dependent and basal activation of [ERS], while the second term corresponds to the basal inactivation of [ERS]. As described by Equation (4), the dynamics of [mTOR] involve the basal and [ERS]-dependent activation, as well as the basal and [Beclin1]-dependent inactivation. The double-negative feedback motif between [Beclin1] and [Caspases] is described in Equations (5) and (6). For the dynamics of [Beclin1] in Equation (4), the first term represents the basal and ERS-dependent activation of [Beclin1], and the second term represents the basal and [Caspases]-dependent inactivation of [Beclin1]. Equation (5) accounts for the dynamics of [Caspases] with two terms, i.e., the basal and [ERS]-dependent activation and the basal and [Beclin1]-dependent inactivation.

Parameters in the model are mostly determined based on the literature (Cloutier et al., 2012; Kapuy et al., 2014) concerning both the simulation results and the experimental data. The initial values for all these variables are set at zero biologically (Cloutier et al., 2012; Kapuy et al., 2014), and the significance of each parameter with its default value is shown in Table 1. The numerical simulations and the bifurcation diagrams of the ODEs are solved by XPPAUT (Ermentrout, 2003).


(1) $$\begin{array}{l} \frac{d[ROS]}{dt}=k_{1}\left[1+S_{1}+d_{\text{α}Syn}\left(\frac{{\left(\frac{\left[\text{α}Syn^{*}\right]}{k_{\text{α}Syn}}\right)}^{4}}{1+{\left(\frac{\left[\text{α}Syn^{*}\right]}{k_{\text{α}Syn}}\right)}^{4}}\right)\right]\\ \;-k_{2}\cdot [ROS]\cdot S_{2} \end{array}$$

(2) $$\begin{array}{l} \frac{d[\text{α}Syn^{*}]}{dt}=k_{3}\cdot [ROS]\cdot S_{3}-k_{4}\cdot \left[\text{α}Syn^{*}\right]\cdot k_{5}\cdot ([Beclin1]\\ \;\cdot [mTOR]) \end{array}$$

(3) $$\begin{array}{l} \frac{d[ERS]}{dt}=k_{6}\cdot \left[\text{α}Syn^{*}\right]\cdot k_{7}\cdot (ERST-[ERS])\\ \;-k_{8}\cdot [ERS] \end{array}$$

(4) $$\begin{array}{l} \frac{d[mTOR]}{dt}=(k_{9}+k_{10}\cdot [ERS])\cdot (mTORT-[mTOR])\\ \;-(k_{11}+k_{12}\cdot [Beclin1])\cdot [mTOR] \end{array}$$

(5) $$\begin{array}{l} \frac{d[Beclin1]}{dt}=\frac{\left(k_{13}+k_{14}\cdot [ERS])\cdot (Beclin1T-[Beclin1]\right)}{\left(Jbe+Beclin1T-[Beclin1]\right)}\\ -\frac{\left(k_{15}+k_{16}\cdot [Caspases]+k_{17}\cdot [mTOR])\cdot [Beclin1\right]}{\left(Jbe+[Beclin1]\right)} \end{array}$$

(6) $$\begin{array}{l} \frac{d[Caspases]}{dt}=\\ \frac{\left(k_{18}+k_{19}\cdot [ERS]+k_{20}\cdot [mTOR])\cdot (CaspasesT-[Caspases]\right)}{Jca+CaspasesT-[Caspases]}\\ -\frac{\left(k_{21}+k_{22}\cdot [Beclin1])\cdot [Caspases\right]}{\left(Jca+[Caspases]\right)} \end{array}$$

**Table 1:** Description of parameters used in models (1)–(6).

**Parameter**	**Significance**	**Value**	**Unit**
*S*_1_	Internal and external oxidative stresses	2	–
*S*_2_	Age-related anti-oxidative mechanisms	1	–
*S*_3_	Influence of genetic damage/mutation	1	–
*d*_α*Syn*_	αSyn*-dependent fractional activation of ROS	15	–
*k*_α*Syn*_	Hill constant of αSyn* aggregation	8.5	μ*M*
*k*_1_	Generation rate of ROS	0.72	μ*Mh*^−1^
*k*_2_	Removal rate constant of ROS	0.72	*h* ^−1^
*k*_3_	Generation rate constant of αSyn*	0.7	*h* ^−1^
*k*_4_	Removal rate constant of αSyn*	0.7	*h* ^−1^
*k*_5_	Beclin1- and mTOR-dependent rate constant of αSyn* degradation	2.7	*h* ^−1^
*k*_6_	αSyn*-dependent rate constant of ERS activation	1	*h* ^−1^
*k*_7_	Basal rate constant of ERS activation	0.5	*h* ^−1^
*k*_8_	Basal rate constant of ERS inactivation	1	*h* ^−1^
*k*_9_	Basal rate of mTOR activation	2	μ*Mh*^−1^
*k*_10_	ERS-dependent rate constant of mTOR activation	10	*h* ^−1^
*k*_11_	Basal rate of mTOR inactivation	0.4	μ*Mh*^−1^
*k*_12_	Beclin1-dependent rate constant of mTOR inactivation	7	*h* ^−1^
*k*_13_	Basal rate of Beclin1 activation	2	μ*Mh*^−1^
*k*_14_	ERS-dependent rate constant of Beclin1 activation	4	*h* ^−1^
*k*_15_	Basal rate of Beclin1 inactivation	1	μ*Mh*^−1^
*k*_16_	Caspases-dependent rate constant of Beclin1 inactivation	10	*h* ^−1^
*k*_17_	mTOR-dependent rate constant of Beclin1 inactivation	0.6	*h* ^−1^
*k*_18_	Basal rate of Caspases inactivation	1	μ*Mh*^−1^
*k*_19_	ERS-dependent rate constant of Caspases activation	2	*h* ^−1^
*k*_20_	mTOR-dependent rate constant of Caspases activation	2	*h* ^−1^
*k*_21_	Basal rate of Caspases inactivation	2	μ*Mh*^−1^
*k*_22_	Beclin1-dependent rate constant of Caspases inactivation	4.5	*h* ^−1^
*Jbe*	Beclin1 Michaelis constant	1	μ*M*
*Jca*	Caspases Michaelis constant	0.04	μ*M*
*ERST*	Total level of ERS	2	μ*M*
*mTROT*	Total level of mTOR	1	μ*M*
*Beclin*1*T*	Total level of Beclin1	1	μ*M*
*CaspasesT*	Total level of Caspases	1	μ*M*

## Results

### Bifurcations analysis under the three stresses

First, we have attempted to understand how every one of the stresses *S*_*i*_ (*i* = 1, 2, 3) changes the activation of [αSyn^*^], [Caspases], and [Beclin1] in the positive-feedback loop between αSyn^*^ and ROS.

**Figure 2:**
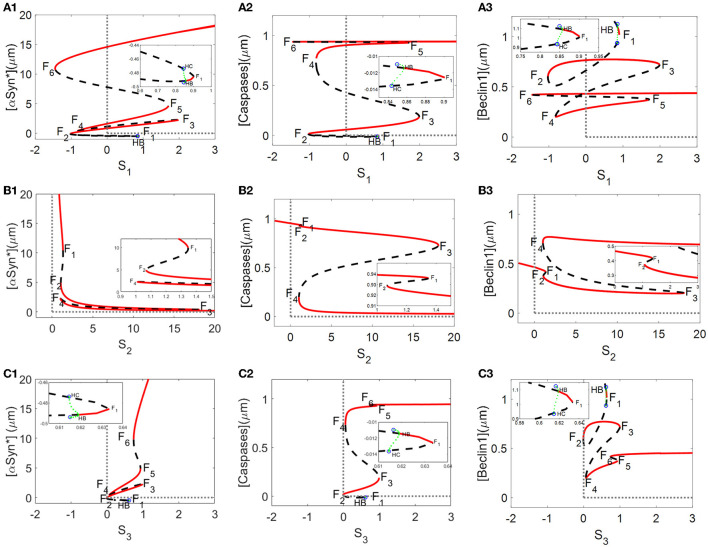
Bifurcation diagrams of [αSyn*], [Caspases], and [Beclin1] for the stresses *S*_1_, *S*_2_, and *S*_3_ [the first lines in **(A–C)**, respectively], where stable and unstable steady states are represented by red solid and black dotted lines, respectively, and *F*_1_–*F*_6_ are fold bifurcation points.

From a dynamic point of view, bifurcation diagrams of the [αSyn^*^] concentration as well as the [Caspases] and [Beclin1] concentrations for the stresses *S*_*i*_ (*i* = 1, 2, 3) were prepared as shown in Figures 2A–C, respectively. There exist multiple stable (red solid lines) and unstable (black dotted lines) bifurcating branches arising from multiple fold points *F*_*i*_(*i* = 1, 2, …, 6), whose locations enable us to decide on the occurrence of tri-stability, bistability, or monostability for the three stresses. In addition, there exist Hopf bifurcation points (HB) on the bifurcation curves for the stresses *S*_1_ and *S*_3_ in Figures 2A, C, respectively. However, only a very small stable limit cycle (green) appears and then disappears *via* saddle homoclinic (HC) bifurcation (see insets in Figures 2A, C).

Here, a relatively large range of values [0, 20] for the stresses *S*_*I*_ (*i* = 1, 2, 3) was set on the ordinate to better display the three coexisting stable steady states. As shown in Figures 2A–C, the tri-stability state dominates the range [1, 1.8] for *S*_1_, being more substantial than the ranges [1.07, 1.34] for *S*_2_ and [1, 1.3] for *S*_3_. It should be pointed out that the results of the three different stable states are from a dynamic standpoint. In fact, the intermediate and high states for [Caspases] almost have the same levels, coinciding with the “0–1” state for [Caspases] in terms of biological significance.

Given that the non-negative stresses in the model are of biological significance, the non-negative half-axis of the (*S*_*i*_, *y*)-plane surrounded by the gray dotted lines is given some focus of attention. All switches between any two stable states are reversible for the stresses *S*_2_ and *S*_3_ (see Figures 2B, C) but irreversible for the stress *S*_1_ due to the fold bifurcation points *F*_2_ and *F*_4_ located in the negative part of the x-axis (see Figure 2A). The reversibility of the stresses *S*_2_ and *S*_3_ guaranteeing arbitrary switching between any two stable states lowers the high [αSyn^*^] concentrations with increasing stress *S*_2_ (see Figure 2B1) or decreasing stress *S*_3_ (see Figure 2C1). Nevertheless, the irreversibility of stress *S*_1_ fails to activate the switch between any two stable states so that the high [αSyn^*^] concentration always remains high (see Figure 2A1).

### Tri-stability initiated from different values of the [αSyn^*^] concentration

**Figure 3:**
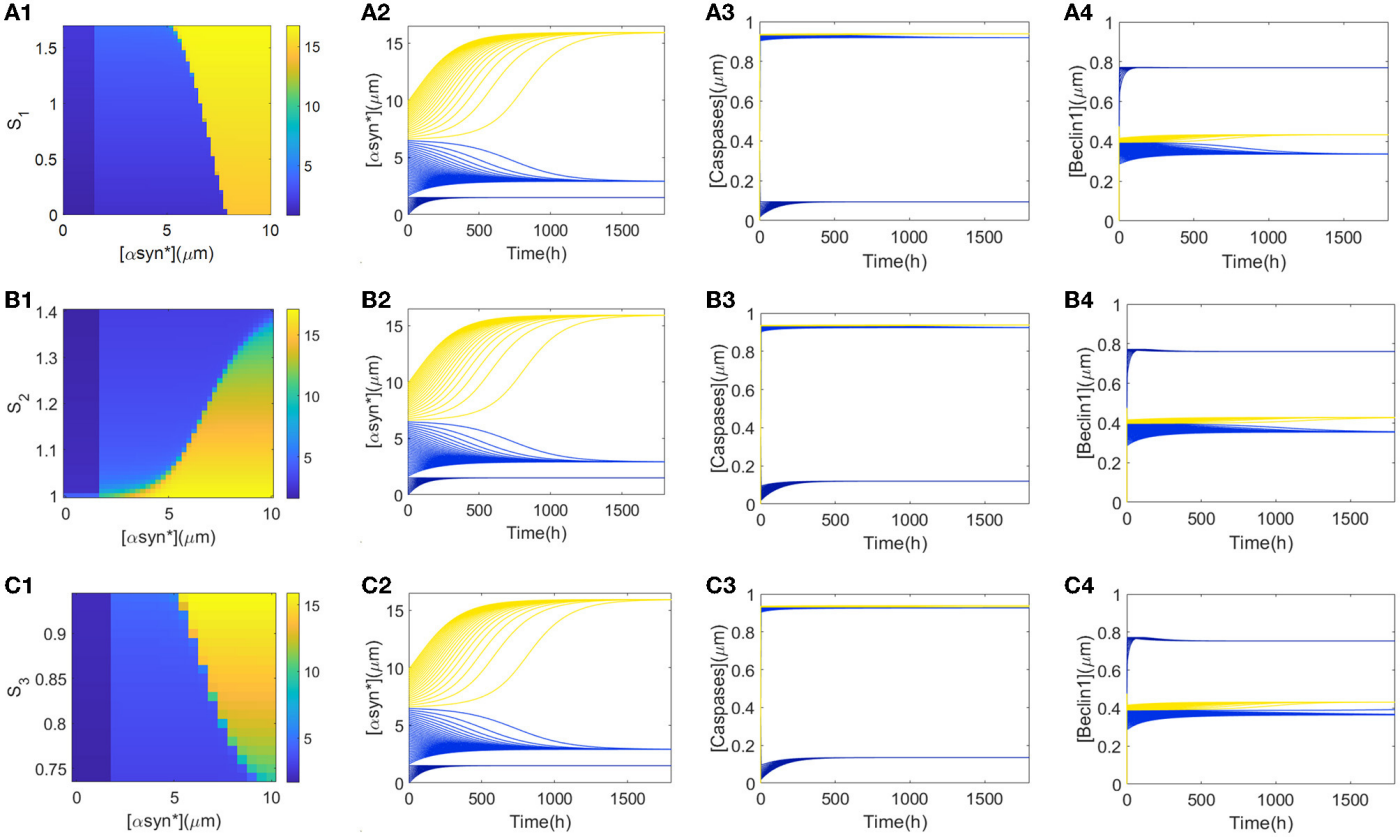
**(A1, B1, C1)** show the three αSyn* levels as a function of the initial values of [αSyn*] (*x*-axis) and *S*_*i*_ (*i* = 1, 2, 3) (*y*-axis). Time series of [αSyn*], [Caspases], and [Beclin1] reach their own three steady-states with the input signal *S*_1_ = 1 **(A2–A4)**, *S*_2_ = 1.2 **(B2–B4)**, and *S*_3_ = 0.9 **(C2–C4)**, respectively.

From Figures 2A–C, it can be deduced that the tri-stability state occupies the ranges of [1, 1.8] for stress *S*_1_, [1.07, 1.34] for *S*_2_, and [1, 1.3] for *S*_3_, respectively. Furthermore, attempts to capture possible states for the initial values of [αSyn^*^] eventually arrive at any of the three steady states for the stresses *S*_*i*_ (*i* = 1, 2, 3) in Figure 3. Within the tri-stable ranges of the three stresses on the vertical axis, all the initiated values of the [αSyn^*^] concentrations (in dark blue, light blue, and yellow) are depicted on the abscissa in Figures 3A1–C1, respectively. To reflect the consequences of different initial values of [αSyn^*^] more intuitively, the time courses for the [αSyn^*^], [Caspases], and [Beclin1] concentrations in the second, third, and fourth columns are presented in Figure 3, respectively, when setting the stress *S*_*i*_ (*i* = 1, 2, 3) at some specific values, i.e., *S*_1_ = 1, *S*_2_ = 1.2, and *S*_3_ = 0.9. In accordance with the results for the bifurcation diagrams in Figures 2A–C, the [αSyn^*^] and [Caspases] concentrations finally reach the lower, middle, and upper stable branches, while the [Beclin1] concentration reaches exactly the upper, lower, and middle stable branches.

Furthermore, explanations regarding the biological significance of the tendencies for the time series of [αSyn^*^], [Caspases], and [Beclin1] concentrations in Figure 3 are now considered. First, small initial values of [αSyn^*^] tend to evoke low [αSyn^*^] concentrations accompanied by high [Beclin1] and low [Caspases] concentrations (dark blue lines in time-series diagrams in Figure 3), which correspond to the health state with a low [αSyn^*^] concentration. From a biological standpoint, Beclin1-induced autophagy takes advantage of the whole process to degrade most of the [αSyn^*^] concentration. Then, medium initial values of [αSyn^*^] lead to a relatively intermediate [αSyn^*^] concentration accompanied by a low [Beclin1] but the intermediate [Caspases] concentrations (light blue lines in time-series diagrams in Figure 3) correspond to critical state with a relatively low [αSyn^*^] concentration. The fact that the [Beclin1] concentration is much lower than the [Caspases] concentration indicates that apoptosis dominates the [αSyn^*^] concentration at this health state. Finally, large initial values of [αSyn^*^] bring about high [αSyn^*^] concentrations, as well as high [Caspases] concentrations, but the intermediate [Beclin1] concentrations (yellow lines in the time-series diagrams in Figure 3) correspond to a disease state due to the high [αSyn^*^] concentrations.

### Tri-stability under the three stresses controlled by the ALP-dependent degradation rate **k**_**5**_

**Figure 4:**
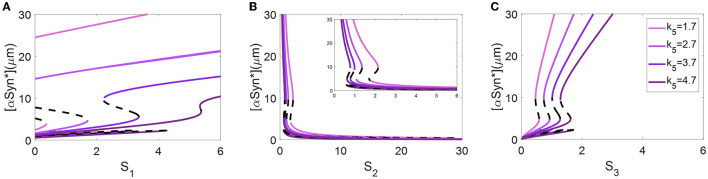
At three different values of *k*_5_, bifurcation diagrams of [αSyn*] for the stress signals *S*_1_ **(A)**, *S*_2_ **(B)**, and *S*_3_ **(C)**, respectively.

Abnormal accumulation of αSyn^*^ is also degraded by ALP *via* both the mTOR-signaling and Beclin1-associated pathways (Shen et al., 2013). In the present model, the ALP-dependent αSyn^*^ degradation rate constant, *k*_5_, bridges the two modules *via* the degradation mechanism of ALP. Here, different values of the parameter *k*_5_ were selected to reveal how the bifurcation curves of αSyn^*^ change with the stress signals *S*_1_, *S*_2_, and *S*_3_ (see Figure 4).

The values of *k*_5_, varying from small to large, resulting in shifting of the bifurcation curves for *S*_1_ and *S*_3_ from left to right (see Figures 4A, C) but for *S*_2_ moves in the opposite direction (see Figure 4B). In particular, the increase in the parameter *k*_5_ results in a change from irreversibility to the reversibility of the bistable switch between the middle and the upper stable states for *S*_1_ in Figure 4A, which further benefits the transition from the upper to the middle stable state. Moreover, the increase in the parameter *k*_5_ significantly lowers the upper stable states to reduce the [αSyn^*^] levels for *S*_1_ in Figure 4A. For example, an increase in *k*_5_ from 2.7 to 3.7 reduces the [αSyn^*^] concentration from 19.27 to 13.27, i.e., a reduction of nearly 31.14%. All of these variations fully support the contention that the aggregation of αSyn^*^ has the possibility to be modulated by the parameter *k*_5_ *via* the ALP degradation pathways. This result may offer a plausible explanation of how the switchover states between irreversibility and reversibility are controlled by ALP which is medicated by mTOR.

### Regions of tri-stable steady states in codimension-2 bifurcation diagrams

The parameter *k*_5_ impacts on the bifurcation curves as well as the switches between the stable steady states. This fact encourages us to identify more globally all the steady-state regions controlled by *k*_5_ through codimension-2 bifurcation diagrams in the (*S*_*i*_, *k*_5_) -planes (*i* = 1, 2, 3 ).

**Figure 5:**
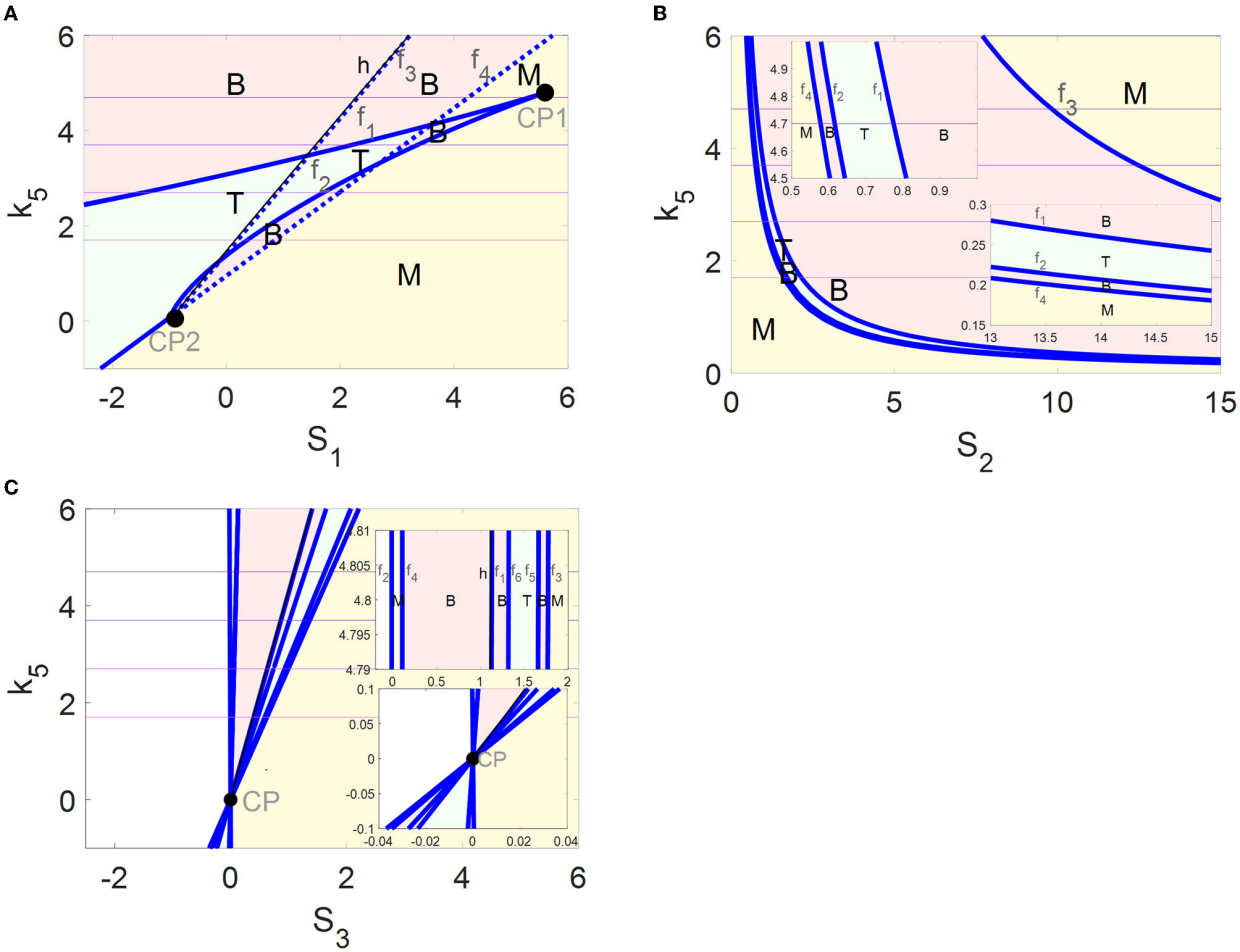
Codimension-2 bifurcation diagrams of αSyn* with respect to *S*_1_ **(A)**, *S*_2_ **(B)**, and *S*_3_ **(C)**, where blue and black curves depict the saddle-node bifurcation points and Hopf bifurcation points, respectively, and T, B, and M denote the tri-stable (green), bistable (pink), and monostable (yellow) states, respectively.

In codimension-2 bifurcation diagrams on the (*S*_1_, *k*_5_)-plane in Figure 5A, four fold bifurcation curves *f*_1_–*f*_4_ (blue) and an Hopf bifurcation curve *h* (black) near *f*_3_ are obtained by the saddle-node bifurcation points *F*_4_, *F*_1_, *F*_5_, and *F*_3_ and the Hopf bifurcation point HB in Figure 2A, respectively. Furthermore, the fold curve *f*_1_ collides with *f*_2_ at the cusp bifurcation points CP1 and *f*_3_ collides with *f*_4_ at CP2. The fold curves *f*_1_, *f*_2_, and *f*_4_ divide the (*S*_1_, *k*_5_)-plane into tri-stable (T, green), bistable (B, pink), and monostable (M, yellow) regions. Here, the stability seems to be unchanged when passing through the fold curve *f*_3_ from left to right. In fact, the number of stable equilibria is added by one *via* fold bifurcation on the fold curve *f*_3_ but reduced by one *via* the Hopf bifurcation on the Hopf curve *h*.

The four fold curves *f*_1_–*f*_4_ in Figure 5B originated from the saddle-node bifurcation points *F*_1_–*F*_4_ (Figure 2B1), where the (*S*_2_, *k*_5_)-plane is divided into regions M, B, T, B, and M from bottom left to top right. In addition, some enlarged views of the upper left and the lower right of the (*S*_2_, *k*_5_)-plane are inserted in Figure 5B.

The saddle-node bifurcation points *F*_1_–*F*_6_ and the Hopf bifurcation point HB in Figure 2C1 form fold bifurcation curves *f*_1_-and the Hopf bifurcation curve *h* in Figure 5C, respectively. It may be pointed out that all the fold curves intersect at one cusp bifurcation point CP. The (*S*_3_, *k*_5_)-plane is divided by *f*_2_–*f*_6_ into regions M, B, T, B, and M from left to right. Here, the stability is still unchanged when passing through the fold curve *f*_1_ and the Hopf curve *h*.

The multi-stability in the regions of M, B, and T with the coexistence of steady states are exhibited more comprehensively by the codimension-2 diagrams in Figure 5. In addition, the dynamics of the tri-stable state in region T should be highlighted, where the healthy state may transform the critical state to avoid transiting to the disease state directly and rapidly. Moreover, the regions of the tri-stability for T that exist in the (*S*_1_, *k*_5_)-plane are much larger than those in the (*S*_2_, *k*_5_)- and the (*S*_3_, *k*_5_)-planes. Furthermore, the irreversible switch transits to the reversible one in the tri-stable region T in the (*S*_1_, *k*_5_)-plane with increasing *k*_5_. Thus, more attention is focused on the tri-stable state for the stress *S*_1_ by the ALP degradation pathways regulated by *k*_5_.

### Fluctuation of the [αSyn^*^] steady-state levels under different initiated conditions

**Figure 6:**
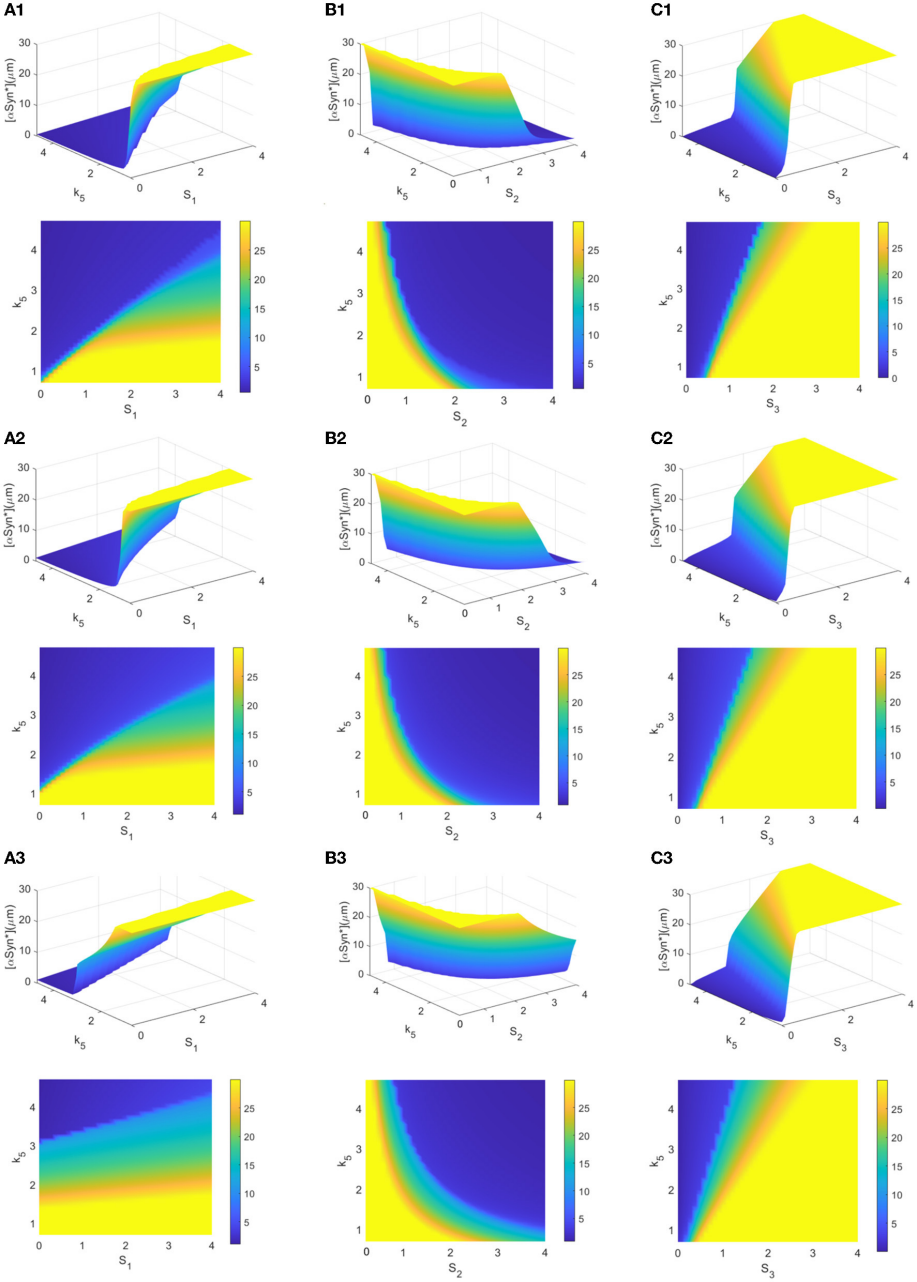
**(A–C)** 3D plots and their contour maps for the [αSyn*] levels at three steady states (blue, green, and yellow) as a function of the stresses *S*_*i*_ and *k*_5_ at the initial values 0, 2, and 10 of αSyn* (rows), respectively.

The choice of initial values is also an important factor in the accumulation of αSyn^*^ as it may lead to the different stable states for tri-stability. From a global perspective, a further survey on the tendency of different initial conditions to affect the steady-state levels of [αSyn^*^] for the three stresses *S*_*i*_ (*i* = 1, 2, 3) and the parameter *k*_5_ was undertaken. Here, the three initial values of [αSyn^*^] were still set at 0, 2, and 10 (see rows 1–3 in Figure 6). In Figure 6, the same results in the 3D and 2D contour maps are presented. The former is to illustrate the variation of the [αSyn^*^] concentration more completely and the latter is to help better understand the response more intuitively. Surfaces of different stable steady states with color bars were constructed for the stresses *S*_*i*_ (*x*-axis) and the parameter *k*_5_ (*y*-axis), and the contour maps are projected onto the planes of (*S*_*i*_, *k*_5_) (*i* = 1, 2, 3) in Figures 6A–C, respectively.

The increasing initial value of [αSyn^*^] from 0 to 2 to 10 elevates the steady-state levels and so inevitably shrinks the regions of the lower stable steady states (blue). Particularly, the extent of shrinking in the (*S*_1_, *k*_5_)-plane in Figure 6A is much sharper than those in the (*S*_2_, *k*_5_)- and (*S*_3_, *k*_5_)-planes in Figures 6B, C. However, the larger *k*_5_ expands the regions of the lower stable steady-state levels for the three stresses *S*_*i*_ (*i* = 1, 2, 3) more to possibly reduce the [αSyn^*^] levels in Figure 6. Especially so for the larger *k*_5_ and the smaller stresses *S*_1_ and *S*_3_ while the larger stress *S*_2_ expands more the regions of the lower stable steady states. In summary, the parameter *k*_5_ contributes to the regulation of the [αSyn^*^] concentration, especially in the case of stress *S*_1_ for the prevention and treatment of PD.

### Robustness of tri-stable state to the parameters of mTOR-related regulations

**Figure 7:**
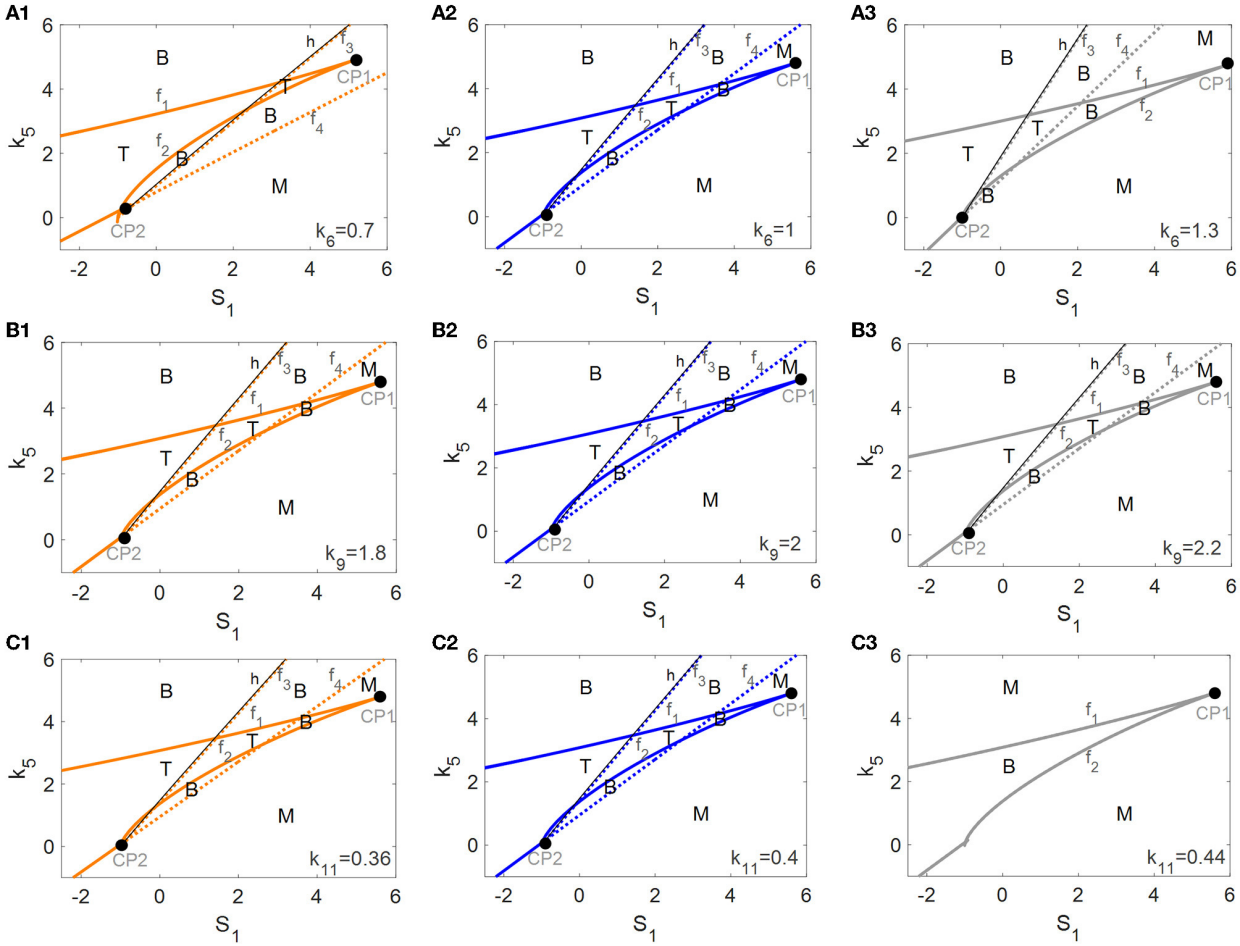
Codimension-2 bifurcation diagrams of *S*_1_ and *k*_5_ for the parameters *k*_6_ **(A)**, *k*_9_ **(B)**, and *k*_11_ **(C)**, where the left, middle, and right columns represent the smaller, the raw, and the larger values for *k*_*i*_ (*i* = 6, 9, 11).

An investigation of the robustness of the tri-stable regions in the (*S*_1_, *k*_5_)-plane to variations in other key parameters was attempted. Here, we consider three important parameters associated with mTOR, i.e., the αSyn^*^-dependent rate constant of ERS activation *k*_6_ as well as the basal rates of mTOR activation and inactivation, *k*_9_ and *k*_11_. The raw values of the parameters *k*_6_ (see Figure 7A), *k*_9_ (see Figure 7B), and *k*_11_ (see Figure 7C) were increased or decreased in the codimension-2 bifurcation diagrams in the (*S*_1_, *k*_5_)-plane in Figure 5A.

With the increase of *k*_6_ from left to right in Figure 7A, the fold bifurcation curves *f*_1_ and *f*_2_ changes little while the fold bifurcation curves *f*_3_ and *f*_4_ shifted to the left. Thus, the tri-stable region T between *f*_1_ and *f*_2_ shrinks while the bistable region B between *f*_1_ and *f*_2_ expands a little. However, *k*_9_ hardly changed any of the bifurcation curves as well as any of the steady-state regions as illustrated in Figure 7B, implying that the dynamic behavior is robust to perturbation of the parameter *k*_9_. Unexpectedly, with the increase of *k*_11_, there are only twofold curves *f*_1_ and *f*_2_ dividing the (*S*_1_, *k*_5_)-plane into monostable and bistable regions, so the tri-stable state is transformed to the bistable state which in turn reverts to the monostable state as shown in Figure 7C.

In summary, the dynamic behavior of the tri-stable state in the (*S*_1_, *k*_5_)-plane changed the most under perturbation of parameter *k*_11_. This result implies that the mTOR-mediated ALP is crucial to maintaining the essential dynamic feature of the tri-stable state for this biological system.

## Discussion

It is widely considered that the aggregation of αSyn^*^ is closely related to the pathogenesis of PD; however, the underlying mechanism is still not fully understood. Indeed, it should be pointed out that the progression of many complex diseases may be divided into three states, i.e., normal state, pre-disease (or tipping point), and disease states (Liu et al., 2017; Liu R. et al., 2019; Liu X. et al., 2019). Especially in neurodegenerative disease (Liu X. et al., 2019; McClellan and King, 2021; Qi et al., 2021), it is quite possible that a critical state may serve as an early-warning signal prior to the rapid switch between healthy and disease states. Therefore, identifying the critical state is crucial and represents a challenge to prevent qualitative deterioration in PD. In this study, we have proposed a mathematical model whereby ALP regulated by mTOR is considered the major protein clearance pathway for degrading the aggregated αSyn^*^; moreover, we have also surveyed the tri-stability dynamics for the three states of PD.

A large amount of aggregated αSyn^*^ which may trigger the spontaneous development of PD may be influenced by multifaceted factors associated with *S*_1_, *S*_2_, and *S*_3_ at the same time, such as exposure to an environmental toxin (external oxidative stress from toxins to increase *S*_1_), advanced age (reduction in the age-related anti-oxidative mechanism of *S*_2_), and a genetic defect (αSyn overexpression or increase in *S*_3_) (Cloutier and Wellstead, 2012). In this research, the key molecules Beclin1 and mTOR in ALP to degrade αSyn^*^ under three different stresses have been highlighted and studied.

The dynamics of tri-stability for the different stress signals *S*_*i*_ (*i* = 1, 2, 3) are captured by codimension-1 bifurcation analysis, in which the lower, middle, and upper stable steady states correspond to the healthy, critical, and disease states in the progression of PD, respectively. Although a small step toward the identification of the intermedium state in PD is taken, the challenges in describing and characterizing faithfully the biological system are expected to be improved in further biodynamic modeling of PD.

It has been established that the bistable switches are irreversible for stress *S*_1_ but reversible for stresses *S*_2_ and *S*_3_. The irreversibility of stress *S*_1_ may ensure that a high concentration of [αSyn^*^] is maintained. From a biological perspective, mitochondria dysfunction plays a crucial role in PD etiopathogenesis, given that it is an important source of *S*_1_ and leads to significantly higher ROS in cells to indirectly accelerate the formation of αSyn^*^ (Cali et al., 2011; Scialo et al., 2017). Moreover, it has been found that the ALP-dependent rate constant of αSyn^*^ degradation, *k*_5_, changes the bistable switch from irreversible to reversible for *S*_1_ and increasing *k*_5_ greatly alters the higher stable states of the αSyn^*^ levels for *S*_1_. It has been confirmed that the ALP degradation pathways offer the possibility to control the aggregation of αSyn^*^ to protect dopaminergic neuron cells from death. More globally, in uncovering all the steady-state regions in the codimension-2 bifurcation diagrams, it was discovered that the regions of tri-stability in the (*S*_1_, *k*_5_)-plane are much larger than those in the (*S*_2_, *k*_5_) and (*S*_3_, *k*_5_)-planes.

Furthermore, it was revealed that mTOR-mediated ALP plays an important role in regulating the degradation of αSyn^*^ based on the robustness of the tri-stable regions in the (*S*_1_, *k*_5_)-*plane* with respect to the three important parameters associated with mTOR. Unexpectedly, the tri-stable dynamic behavior vanished under the disturbance of the *k*_11_ parameter, implying that mTOR-mediated ALP is important to maintaining the essential dynamic features of tri-stability for biological systems. In addition, it is well-known that the function of mTOR signaling is of great importance in restoring neuron death induced by toxins, such as the huge accumulation of αSyn^*^ in PD (Ebrahimi-Fakhari et al., 2014; Lan et al., 2017).

In conclusion, the clearance mechanism of ALP plays an important role in tri-stability in our model where mTOR-mediated ALP degrades αSyn^*^. The model links experimental and theoretical biology to realize a more comprehensive understanding of the precise regulatory mechanisms of the degradation of αSyn^*^ by ALP and mediated by mTOR. In future, the essential properties of tri-stability may be applied to experiments and studies of PD, and relevant molecular aspects should be considered for biomathematical modeling and dynamic analysis of PD.

The intermediate state as a barrier prevents the system from switching from the lower to the upper steady state directly. Thus, the critical state (the intermediate state) in tri-stability is an alert warning for avoiding more aggregation of αSyn^*^ and causing PD, thus treatment for the critical state in neuron cells will be vital for modeling PD dynamic networks. Our study may provide a promising avenue for further experiments and simulations of the degradation mechanisms for dynamic modeling in PD. This research may ultimately lead to novel therapeutic approaches for the treatment of PD.

## Data availability statement

The original contributions presented in the study are included in the article/supplementary material, further inquiries can be directed to the corresponding author.

## Author contributions

BY was responsible for manuscript development, model concept, and writing the manuscript. ZY supervised the study design and manuscript development. LH collaborated on manuscript development and concept. All authors have read and approved the final manuscript.
